# Predictive Value of Arterial Blood Lactic Acid Concentration on the Risk of in-Hospital All-Cause Death in Patients with Acute Heart Failure

**DOI:** 10.1155/2022/7644535

**Published:** 2022-11-16

**Authors:** Weiwei Hu, Lei Yuan, Xiaotong Wang, Baohe Zang, Yang Zhang, Xianliang Yan, Wenjing Zhao, Yali Chao

**Affiliations:** ^1^Department of Critical Care Medicine, The Affiliated Hospital of Xuzhou Medical University, Xuzhou 221000, Jiangsu, China; ^2^Department of Interventional Vascular Surgery, Xuzhou Cancer Hospital, Xuzhou 221005, Jiangsu, China; ^3^Department of Cardiology, The Affiliated Hospital of Xuzhou Medical University, Xuzhou 221000, Jiangsu, China; ^4^Department of Emergency Medicine, The Affiliated Hospital of Xuzhou Medical University, Xuzhou 221000, Jiangsu, China

## Abstract

The study aims to examine the predictive value of arterial blood lactic acid concentration for in-hospital all-cause mortality in the intensive care unit (ICU) for patients with acute heart failure (AHF). We retrospectively analyzed the clinical data of 7558 AHF patients in the Medical Information Mart for Intensive Care IV (MIMIC-IV) database. The exposure variable of the present study was arterial blood lactic acid concentration and the outcome variable was in-hospital all-cause death. The patients were divided into those who survived (*n* = 6792) and those who died (*n* = 766). The multivariate logistic regression model, restricted cubic spline (RCS) plot, and subgroup analysis were used to evaluate the association between lactic acid and in-hospital all-cause mortality. In addition, receiver operating curve (ROC) analysis also was performed. Finally, we further explore the association between NT-proBNP and lactic acid and in-hospital all-cause mortality. Compared with the lowest quartiles, the odds ratios with 95% confidence intervals for in-hospital all-cause mortality across the quartiles were 1.46 (1.07–2.00), 1.48 (1.09–2.00), and 2.36 (1.73–3.22) for lactic acid, and in-hospital all-cause mortality was gradually increased with lactic acid levels increasing (*P* for trend <0.05). The RCS plot revealed a positive and linear connection between lactic acid and in-hospital all-cause mortality. A combination of lactic acid concentration and the Simplified Acute Physiology Score (SAPS) II may improve the predictive value of in-hospital all-cause mortality in patients with AHF (AUC = 0.696). Among subgroups, respiratory failure interacted with an association between lactic acid and in-hospital all-cause mortality (*P* for interaction <0.05). The correlation heatmap revealed that NT-proBNP was positively correlated with lactic acid (*r* = 0.07) and positively correlated with in-hospital all-cause mortality (*r* = 0.18). There was an inverse L-shaped curve relationship between NT-proBNP and in-hospital all-cause mortality, respectively. Mediation analysis suggested that a positive relationship between lactic acid and in-hospital all-cause death was mediated by NT-proBNP. For AHF patients in the ICU, the arterial blood lactic acid concentration during hospitalization was a significant independent predictor of in-hospital all-cause mortality. The combination of lactic acid and SAPS II can improve the predictive value of the risk of in-hospital all-cause mortality in patients with AHF.

## 1. Introduction

Acute heart failure (AHF) is a frequent clinical critical condition that is mostly represented by cardiogenic shock and acute pulmonary edema [1]. Due to the quick onset and progression of AHF, it is critical to provide prompt and appropriate treatment [2]. Thus, quick and precise evaluation and treatment of AHF patients may improve patient outcomes. People have begun to pay more attention to the prediction of early AHF death as a result of the recent increase in AHF patient mortality [3]. At the moment, N-terminal B-type natriuretic peptide precursor (NT-proBNP) is a frequently utilized indicator for clinical assessment of heart failure patients' prognosis, although its sensitivity and specificity are restricted [4]. Therefore, identifying biomarkers with a high degree of sensitivity and specificity is critical for assessing the prognosis of patients with AHF.

Previous research has shown that elevated arterial blood lactic acid concentration is connected with an increased risk of morbidity and death in some severe illnesses, implying that lactic acid is a crucial predictor of prognosis in critically sick patients [5]. Research by Robert et al. has shown that elevated blood lactic acid on admission is common in AHF patients and is associated with markers of a worse prognosis [6]. Some studies also suggest that lactic acid can predict the prognosis of patients with AHF [7–9]. The association between the levels of lactic acid and nosocomial all-cause mortality in patients with AHF, especially in the critical care unit, has yet to be determined. In critical care medicine, the Medical Information Mart for Intensive Care IV (MIMIC-IV) database is a comprehensive single-center database that contains demographic and clinical data on all patients admitted to the ICU between 2008 and 2019 [10]. In this study, we searched for and analyzed clinical data from AHF patients admitted to the MIMIC-IV database to determine if lactic acid is associated with in-hospital all-cause mortality in patients with AHF after admission.

## 2. Materials and Methods

### 2.1. Data Source

The MIMIC-IV database, a publicly available critical care database, was used for all the research [11]. Between 2008 and 2019, the MIMIC-IV database contains clinical data on patients admitted to the intensive care unit (ICU) at Beth Israel Deaconess Medical Center (BIDMC), including demographics and infections, birth and death, ICU admission and discharge, vital signs, laboratory data, and body fluid balance, as well as reports, medications, and nursing records. After passing the Protection of Human Research Participants Examination and the NIH online training course, we were able to access the MIMIC-IV database and harvest data (certification number: 50141059). SQL (structured query language) was used to get all data from the MIMIC-IV database. In addition, we also included AHF patients from the Department of Critical Care Medicine, Affiliated Hospital of Xuzhou Medical University between 2018 and 2022 to verify the association between arterial blood lactic acid concentration and in-hospital all-cause mortality.

### 2.2. Study Population

We retrieved and extracted patients with AHF from the MIMIC-IV database between 2008 and 2019. The inclusion criteria were as follows: 10919 AHF patients were enrolled, of which 2679 AHF patients were excluded due to not being admitted to the hospital and ICU for the first time. Of the remaining 8240 AHF patients, 622 AHF patients were excluded because of the age of less than 18 and severely missing data. Finally, 7558 AHF patients were included in the present study. Medical records of the patient's baseline data including the patient's age, gender, ethnicity, body mass index (BMI), height, weight, systolic blood pressure (SBP), diastolic blood pressure (DBP), serum anion gap (SAP), bicarbonate, blood urea nitrogen (BNU), calcium, chloride, serum creatinine (Scr), glucose, serum sodium, serum potassium, hematocrit, hemoglobin (Hb), platelet (Plt), red blood cell (RBC), C reactive protein (CRP), length of stay (LOS), albumin, lactic acid, white blood cell (WBC), N terminal pro B type natriuretic peptide (NT-proBNP), Simplified Acute Physiology Score (SAPS) II, sequential organ failure assessment (SOFA) score, and history of drug use of cardiotonic, nitro-glycerine, furosemide, hypoglycaemic, and infection-fighting. In addition, we also collected patient-related complications such as chronic obstructive pulmonary disease (COPD), hypertension, diabetes mellitus (DM), malignancy, respiratory failure (RF), septicaemia, ventricular fibrillation (VF), atrial fibrillation (AF), liver cirrhosis, acute myocardial infarction (AMI), chronic kidney disease (CKD). The mean lactic acid levels throughout hospitalization were utilized and the other blood test results were all the results of the patient's initial examination in the ICU. When patients were hospitalized in the ICU more than once, the clinical data from the initial admission were used. In addition, according to the above inclusion and exclusion criteria, we enrolled 322 patients with AHF in the Intensive Care Unit of the Affiliated Hospital of Xuzhou Medical University.

### 2.3. Grouping and the End-Point

AHF patients were grouped according to survivors and deaths. The end-point was defined as in-hospital all-cause death that occurred in patients with AHF.

### 2.4. Statistical Analyses

Continuous variables were represented as mean ± standard deviation or median (25th quartile, 75th quartile) and categorical variables are presented as a percentage. Continuous variables were compared between groups using the *T*-test and Wilcoxon test, and categorical comparisons between groups were performed using the *X*2 test. Model 1 was not adjusted for variables. Model 2 was adjusted for age, gender, ethnicity. Model 3 was adjusted for age, ethnicity, BMI, weight, SBP, the history of AF, liver cirrhosis, AMI, CKD, RF, COPD, VF, DM, and septicaemia, SAP, bicarbonate, BUN, serum calcium, serum creatinine, glucose, hematocrit, Hb, Plt, RBC, CRP, LOS, albumin, WBC, NT-proBNP, cardiotonic drugs, nitro-glycerine drugs, furosemide drugs, infection-fighting drugs, SAPS II, and SOFA score. The restricted cubic spline (RCS) was used to evaluate the association between lactic acid and in-hospital all-cause mortality and the area under the receiver operating characteristic curve (AUC) was performed to assess the predictive value of lactic acid on the risk of in-hospital death. The subgroup analysis was performed by stratified multivariate logistic regression analysis under different COPD, hypertension, DM, RF, septicaemia, VF, AF, AMI, and CKD. The purpose of mediation analysis is to determine which factors play a mediating role in the relationship between the exposure and the disease. All statistical analyses were performed using the software Stata 15 and *R* 4.13. All *P* value <0.05 was significant.

## 3. Results

### 3.1. Baseline Characteristics

**Figure 1:**
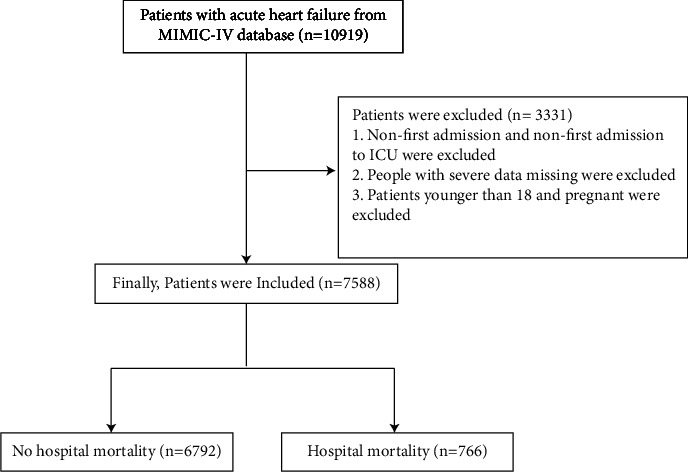
Flow chart of patient selection included in this study. Abbreviations: MIMIC-IV, The medical information mart for intensive care IV.

According to inclusion and exclusion criteria, this study included a total of 7558 AHF patients, with 6792 patients in the survival group and 766 patients in the death group. There is a flow diagram for our screening and grouping study shown in Figure 1. Compared with the survival group, patients in the death group were older and more likely to have liver cirrhosis, AMI, CKD, malignancy, RF, COPD, DM, and septicaemia, but less likely to have hypertension. In addition, the death group had lower levels of SBP, bicarbonate, calcium, hematocrit, RBC, hemoglobin, platelets and albumin, higher levels of SAP, glucose, CRP, BUN, Scr, WBC, NT-proBNP, SAPS score II and SOFA score, and longer LOS. Meanwhile, patients in death group also were easier to use cardiotropic drugs, vasoactive drugs, and infection-fighting drugs (*P* < 0.05, Table 1). The basic characteristics of the 322 participants from ICU of the Affiliated Hospital of Xuzhou Medical University are shown in Supplementary Table 1.

### 3.2. Association between Lactic Acid and the Risk of In-Hospital All-Cause Death

**Figure 2:**
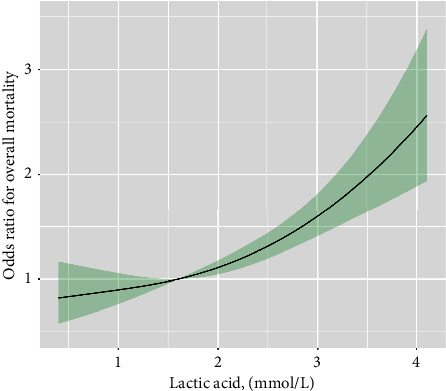
Restricted cubic spline plots of associations between lactic acid levels and in-hospital all-cause mortality. Note: Analyses were adjusted for age, ethnicity, body mass index, weight, systolic blood pressure, the history of atrial fibrillation, liver cirrhosis, acute myocardial infarction, chronic kidney disease, respiratory failure, chronic obstructive pulmonary disease, ventricular fibrillation, diabetes mellitus, septicaemia, serum anion gap, bicarbonate, blood urea nitrogen, calcium, serum creatinine, glucose, hematocrit, hemoglobin, platelet, red blood cells, C reactive protein, length of stay, albumin, white blood cells, N terminal pro B type natriuretic peptide, cardiotonic drugs, nitroglycerin drugs, furosemide drugs, infection-fighting drugs, simplified Acute Physiology Score II, and sequential organ failure assessment. The solid line and dashed line represent the log-transformed odds ratios and corresponding 95% confidence intervals.

In a fully adjusted model III, compared with the lowest quartiles, the odds ratios (ORs) with 95% confidence intervals (CIs) for in-hospital all-cause death across the quartiles were 1.46 (1.07–2.00), 1.48 (1.09–2.00), and 2.36 (1.73–3.22) for lactic acid (Table 2). The RCS also showed a linear relationship and a positive correlation between lactic acid and in-hospital all-cause mortality (Figure 2). Using data from patients with AHF in ICU of the Affiliated Hospital of Xuzhou Medical University for validation, we found that the ORs with 95% CIs for in-hospital all-cause death across the quartiles were 0.52 (0.31, 0.87), 0.73 (0.43, 1.22), and 1.13 (0.68, 1.86) for lactic acid, compared with the lowest quartiles (Supplementary Table II). Meanwhile, there was also a linear correlation between lactic acid and in-hospital all-cause mortality (Supplementary Figure 1).

### 3.3. Diagnostic Value of Lactic Acid

**Figure 3:**
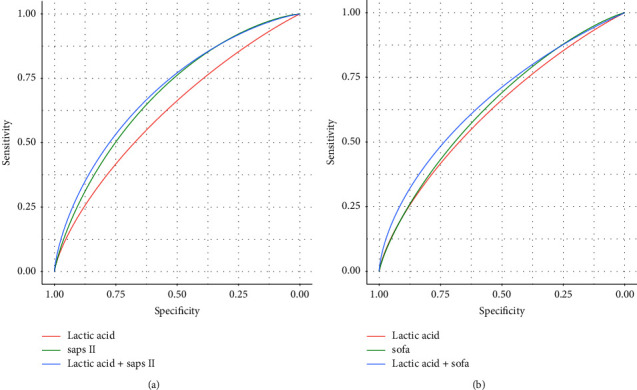
Receiver operating characteristic curve for predicting the risk of in-hospital all-cause death in patients with acute heart failure. (a), The AUC of lactic acid = 0.616, with a sensitivity of 52.22% and a specificity of 63.35%. The AUC of SAPS II = 0.681, with a sensitivity of 67.75% and a specificity of 57.80%. The AUC of lactic acid combined with SAPS II = 0.696, with a sensitivity of 61.88% and a specificity of 65.89%. (b). The AUC of SOFA score = 0.646, with a sensitivity of 64.60% and a specificity of 63.74%. The AUC of lactic acid combined with SOFA score = 0.661, with a sensitivity of 76.37% and a specificity of 48.13%. Note: The *x* axis represents the false-positive rate, while the *y* axis shows the true-positive rate. Abbreviations: ROC, Receiver operating characteristic; AUC, the area under ROC curve; SAPS II, Simplified acute physiology score II; and SOFA, Sequential organ failure assessment.

As illustrated in Figure 3(a), the receiver operator characteristic curve constructed by the indicator variable lactic acid was able to predict in-hospital all-cause mortality and the AUC was 0.616 (sensitivity: 52.22% and specificity: 63.35%). The AUC of the lactic acid plus the SAPS II for the diagnosis of in-hospital all-cause death was 0.696, and the sensitivity and specificity were 61.88% and 65.89%, respectively. In addition, the AUC of the SOFA score in predicting the risk of in-hospital death was 0.646, while the AUC of lactic acid combined with the SOFA score was 0.661 (Figure 3(b)).

### 3.4. Subgroup Analysis

The present study based on a subgroup analysis of COPD, DM, hypertension, RF, septicaemia, VF, AF, AMI, and CKD, showed the relationship between arterial blood lactic acid and in-hospital all-cause death. Among subgroups, RF interacted with the association between arterial blood lactic acid and in-hospital all-cause death (*P* for interaction <0.05). Except for the subgroup of RF, the interaction of arterial blood lactic acid for COPD, DM, hypertension, septicaemia, VF, AF, AMI, and CKD with in-hospital all-cause death were not significant (Table 3).

### 3.5. Association between NT-proBNP, Lactic Acid, and the Risk of In-Hospital All-Cause Death

**Figure 4:**
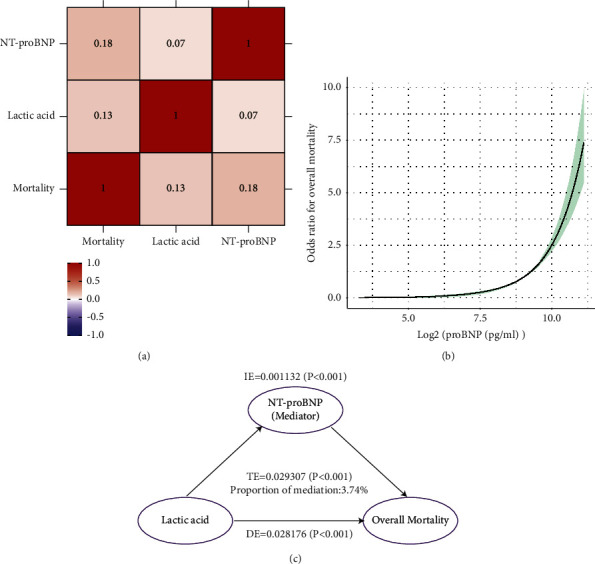
The association between NT-proBNP, lactic acid, and the risk of in-hospital all-cause death. (a) Correlation matrix of NT-proBNP, lactic acid, and the risk of in-hospital all-cause death. (b) Restricted cubic spline plots of associations between NT-proBNP levels and in-hospital all-cause mortality. Note: Analyses were adjusted for age, ethnicity, body mass index, weight, systolic blood pressure, the history of atrial fibrillation, liver cirrhosis, acute myocardial infarction, chronic kidney disease, respiratory failure, chronic obstructive pulmonary disease, ventricular fibrillation, diabetes mellitus, septicaemia, anion gap, bicarbonate, blood urea nitrogen, calcium, serum creatinine, glucose, hematocrit, hemoglobin, platelet, red blood cells, C reactive protein, length of stay, albumin, white blood cells, lactic acid, cardiotonic drugs, nitroglycerin drugs, furosemide drugs, infection-fighting drugs, Simplified Acute Physiology Score II, and sequential organ failure assessment. The solid line and dashed line represent the log-transformed odds ratios and corresponding 95% confidence intervals. (c) Mediation analysis of NT-proBNP on the interaction between lactic acid levels and in-hospital all-cause death. Notes: Mediation models of NT-proBNP, lactic acid, and in-hospital all-cause death in data: direct effect (TE = 0.029307; *P* < 0.001) of lactic acid (exposure) toward in-hospital all-cause death (outcome), and NT-proBNP medication proportion is 5.9%; indirect effect (IE = 0.001132; *P* < 0.001) of lactic acid (exposure) toward NT-proBNP (mediator) and effect obesity (DE = 0.028176; *P* < 0.001), from NT-proBNP (mediator) toward in-hospital all-cause death (outcome).

In the full adjusted multivariate logistic regression model, NT-proBNP was an independent predictor of in-hospital all-cause death and compared with the lowest quartile, the ORs with 95%CIs for association of NT-proBNP with in-hospital all-cause death in the fourth quartiles was 13.39 (9.52–18.83) (*P* < 0.001) (Table 4). The correlation heatmap of NT-proBNP, lactic acid, and in-hospital all-cause death revealed that NT-proBNP was positively related with lactic acid (*r* = 0.07) and NT-proBNP were positively related with in-hospital all-cause mortality (*r* = 0.18) (Figure 4(a)). The RCS showed an inverse L-shaped curve relationship between NT-proBNP and in-hospital all-cause mortality (Figure 4(b)). Mediation analysis was undertaken to assess if the association between NT-proBNP and in-hospital all-cause death was mediated by lactic acid. The NT-proBNP was estimated to explain 3.74% of the association between the lactic acid levels and in-hospital all-cause death (IE: *β* = 0.001132, *P* < 0.001; TE: *β* = 0.029307, *P* < 0.001; and DE: *β* = 0.028176, *P* < 0.001) (Figure 4(c)).

## 4. Discussion

AHF is a group of clinical syndromes characterized by abnormal cardiac structure and function, resulting in a sharp decline in cardiac displacement, hypoperfusion of tissues and organs, and acute blood stasis, with higher mortality and rehospitalization rates than that of chronic heart failure [12]. Lactic acid levels in the circulation are an important indicator of the availability of oxygen to the tissues, metabolism, and perfusion of the body's cells [13]. A rise in arterial blood lactic acid concentrations occurs when the body is under oxygen-depleted conditions. As a result, the arterial blood lactic acid levels may be utilized in clinical practice to evaluate the microcirculation function of tissues and organs [14]. Findings from this study show that high lactic acid levels are a significant risk factor for in-hospital all-cause mortality in patients with AHF. Our results are consistent with previous studies, suggesting that arterial blood lactic acid concentration plays an important role in determining the prognosis of heart failure [5].

Lactic acid levels are associated with the prognosis of a variety of diseases [15–17]. We performed a subgroup analysis by disease type, allowing for possible interference from other comorbidities. Finally, elevated lactic acid levels were linked with AHF mortality independent of the presence or absence of COPD, DM, hypertension, RF, septicaemia, VF, AF, AMI, and CKD. Notably, lactic acid levels in the first three quartiles were not significantly associated with all-cause mortality in patients without respiratory failure, whereas there was a significant association between lactic acid levels and mortality in patients with respiratory failure. Previous studies have demonstrated that lactic acid levels are associated with outcomes in patients with RF [18]. This suggests that the prognosis of respiratory failure is more sensitive to arterial lactic acid levels than AHF, however, after the exclusion of RF, arterial lactic acid at Q4 levels is still significantly associated with all-cause death in AHF. For this reason, we expected that higher lactic acid levels were related to an increased risk of all-cause mortality within 28 days of admission for AHF patients.

Patients with AHF are unable to provide appropriate oxygenation and perfusion of tissues and organs owing to infection, decreased cardiac function, coronary artery stenosis, and other factors, increasing in anaerobic glycolysis and lactic acid build-up [19]. Narang et al. revealed that cardiac index decreases when lactic acid levels rise in individuals with acute decompensated heart failure [7]. Li et al. found that as lactic acid levels climbed, in-hospital mortality in patients with heart failure increased proportionately [20]. Kawase et al. discovered that blood lactic acid levels were linked to in-hospital mortality in patients with acute heart failure without acute coronary syndrome, and that serum lactic acid levels might assist in predicting the risk of early death in individuals with acute decompensated heart failure [21]. In addition, lactic acid levels have been proven to be an independent predictor of death in hospitalized patients with heart failure. When the lactic acid levels in acutely decompensated heart failure patients exceeds 3.2 mmol/L, the hospital mortality rate increases [22, 23]. Once significant amounts of blood lactic acid and its metabolites are stored in the body, patients will gradually progress into severe metabolic acidosis, which will eventually result in PH decline, hyperkalaemia, and a decrease in the ventricular fibrillation threshold, further increasing the incidence of sudden cardiac death [24]. In the study of Kapur et al. lactic acid levels were also added to the classification criteria for cardiogenic shock. Therefore, we speculate that blood lactic acid is a very useful clinical parameter for assessing the severity and prognosis of AHF [25]. The association between NT-proBNP and in-hospital all-cause rate was finally identified as a nearly inverse L-shaped curve. In addition, the effect of lactic acid on in-hospital all-cause mortality was influenced by NT-proBNP. As mentioned above, higher lactic acid concentrations were associated with higher in-hospital all-cause mortality in patients with AHF. A recent study showed that NT-proBNP is independent predictors of all-cause and cardiovascular-related mortality in patients with acute decompensated heart failure [26]. Therefore, further research is needed to determine how lactic acid and BNP are related.

In conclusion, it is important to note that in patients with AHF, dynamic arterial blood lactic acid levels, which may help determine the illness severity and prognosis, can be found. In addition, the study had a number of flaws. To begin with, this is a retrospective study, which creates a bias in the selection of subjects. Second, the sample size was not large enough, especially for the death group. After the subgroup analysis, the sample size of each subgroup was different, for the analysis of some concomitant diseases, the sample size was too small to produce potential bias, and our results may have been impacted by other unknown variables, even though we adjusted for certain factors.

## 5. Conclusion

The levels of arterial blood lactic acid were an independent predictor of all-cause death of admission for AHF patients in the ICU. The combination of lactic acid and SAPS II may increase the accuracy of predicting the likelihood of in-hospital all-cause death in AHF patients. There was a positive relationship between lactic acid and in-hospital all-cause death was mediated by NT-proBNP.

## Figures and Tables

**Table 1:** Baseline characteristics of the AHF patients on admission.

Variables	Overall (*n* = 7558)	Survival group (*n* = 6792)	Death group (*n* = 766)	*P* value
Age, years	68.52 ± 13.56	67.98 ± 13.58	73.29 ± 12.43	<0.001
Female, (%)	3358 (44.4%)	3036 (44.7%)	322 (42.0%)	0.171
Ethnicity, (%)			<0.001	
White	5113 (67.7%)	4618 (68.0%)	495 (64.6%)	
Black	1026 (13.6%)	981 (14.4%)	45 (5.9%)	
Other	1419 (18.8%)	1193 (17.6%)	226 (29.5%)	
BMI, kg/m^2^	30.04 ± 7.89	30.18 ± 7.93	28.78 ± 7.45	<0.001
Height, cm	168.08 ± 11.14	168.08 ± 11.18	168.13 ± 10.83	0.889
Weight, kg	84.98 ± 23.57	85.38 ± 23.73	81.36 ± 21.78	<0.001
SBP, mmHg	114.01 ± 14.83	114.76 ± 13.93	107.33 ± 19.98	<0.001
DBP, mmHg	57.97 ± 9.68	58.01 ± 9.36	57.56 ± 12.15	0.218
AF, (%)	3513 (46.5%)	3039 (44.7%)	474 (61.9%)	<0.001
Liver cirrhosis, (%)	152 (2.0%)	124 (1.8%)	28 (3.7%)	0.001
AMI, (%)	725 (9.6%)	584 (8.6%)	141 (18.4%)	<0.001
CKD, (%)	3084 (40.8%)	2708 (39.9%)	376 (49.1%)	<0.001
Malignancy, (%)	20 (0.3%)	16 (0.2%)	4 (0.5%)	0.274
RF, (%)	2203 (29.1%)	1644 (24.2%)	559 (73.0%)	<0.001
COPD, (%)	750 (9.9%)	641 (9.4%)	109 (14.2%)	<0.001
VF, (%)	145 (1.9%)	94 (1.4%)	51 (6.7%)	<0.001
Hypertension, (%)	418 (5.5%)	378 (5.6%)	40 (5.2%)	0.756
DM, (%)	1421 (18.8%)	1251 (18.4%)	170 (22.2%)	0.013
Septicaemia, (%)	833 (11.0%)	703 (10.4%)	130 (17.0%)	<0.001
SAP, mmol/L	14.81 ± 3.53	14.69 ± 3.46	15.89 ± 3.92	<0.001
Bicarbonate, mmol/L	26.48 ± 5.26	26.64 ± 5.15	25.05 ± 5.98	<0.001
BUN, mg/dl	30.00 (20.00, 47.00)	29.00 (20.00, 47.00)	37.00 (24.00, 54.00)	<0.001
Calcium, mg/dl	8.72 ± 0.66	8.74 ± 0.65	8.53 ± 0.69	<0.001
Chloride, mmol/L	100.00 ± 6.18	99.96 ± 6.12	100.31 ± 6.73	0.138
Scr, mg/dL	1.30 (0.90, 2.00)	1.30 (0.90, 2.00)	1.50 (1.00, 2.38)	<0.001
Glucose, mg/dL	123.20 (102.00, 157.00)	123.00 (102.00, 156.00)	129.00 (103.00, 167.00)	0.004
Sodium, mmol/L	138.27 ± 4.66	138.26 ± 4.62	138.42 ± 4.95	0.355
Potassium, mmol/L	4.22 ± 0.58	4.22 ± 0.58	4.25 ± 0.58	0.173
Hematocrit, %	31.65 ± 6.08	31.71 ± 6.09	31.14 ± 6.03	0.014
Hb, g/dl	10.19 ± 2.07	10.21 ± 2.08	10.01 ± 2.03	0.013
Plt, ×10^9^/L	208.00 (151.00, 273.00)	211.00 (154.00, 276.00)	186.00 (121.25, 253.00)	<0.001
RBC, ×10^9^/L	3.49 ± 0.72	3.50 ± 0.72	3.42 ± 0.71	0.008
CRP, mg/L	46.34 (20.37, 77.20)	42.96 (18.80, 72.70)	76.85 (49.75, 108.30)	<0.001
LOS, days	3.22 (1.70, 6.14)	3.15 (1.69, 5.94)	4.12 (1.90, 8.20)	<0.001
Albumin, g/dL	3.48 ± 0.64	3.51 ± 0.62	3.14 ± 0.68	<0.001
Lactic acid, mmol/L	1.86 ± 0.97	1.82 ± 0.94	2.20 ± 1.12	<0.001
WBC, ×10^9^/L	9.00 (6.70, 11.90)	8.80 (6.70, 11.60)	11.00 (7.80, 15.00)	<0.001
NT-proBNP, pg/ml	8366.49 (3589.00, 11382.00)	7832.12 (3215, 10513.47)	14028.77 (12100.78, 15956.01)	<0.001
Cardiotonic, (%)	2288 (30.3%)	2030 (29.9%)	258 (33.7%)	0.034
Nitro-glycerine, (%)	4416 (58.4%)	4131 (60.8%)	285 (37.2%)	<0.001
Furosemide, (%)	7315 (96.8%)	6609 (97.3%)	706 (92.2%)	<0.001
Hypoglycaemic, (%)	1422 (18.8%)	1272 (18.7%)	150 (19.6%)	0.566
Infection-fighting, (%)	892 (11.8%)	630 (9.3%)	262 (34.2%)	<0.001
SAPS II score	39.00 (32.00, 48.00)	38.00 (31.00, 47.00)	46.00 (37.00, 57.00)	<0.001
SOFA score	2.00 (1.00, 5.00)	2.00 (1.00, 5.00)	3.00 (1.00, 6.00)	<0.001

Abbreviations: AHF, acute heart failure; BMI, body mass index; SBP, systolic blood pressure; DBP, diastolic blood pressure; AF, atrial fibrillation; AMI, acute myocardial infarction; CKD, chronic kidney disease; RF, respiratory failure; VF, ventricular fibrillation; SAP, serum anion gap; BUN, blood urea nitrogen; Scr, serum creatinine; Hb, hemoglobin; Plt, platelet; RBC, red blood cell; CRP, C-reactive protein; SOFA, sequential organ failure assessment. LOS, length of stay (LOS); WBC, white blood cell; NT-proBNP, N terminal pro B type natriuretic peptide; and SAPS II score, simplified acute physiology score II.

**Table 2:** Association between lactic acid levels and the risk of in-hospital deaths.

Lactic acid	Model 1	Model 2	Model 3
Q1, mmol/L	Ref.	Ref.	Ref.
Q2, mmol/L	1.63 (1.26–2.11) ^*∗∗∗*^	1.56 (1.21–2.03) ^*∗∗*^	1.44 (1.07–1.93) ^*∗*^
Q3, mmol/L	1.76 (1.36–2.29) ^*∗∗∗*^	1.66 (1.28–2.16) ^*∗∗∗*^	1.46 (1.08–1.98) ^*∗*^
Q4, mmol/L	2.75 (2.14–3.55) ^*∗∗∗*^	2.69 (2.08–3.48) ^*∗∗∗*^	2.41 (1.78–3.27) ^*∗∗∗*^
*P* For trend	<0.001	<0.001	<0.001

Abbreviation: Q1, 0.3–1.1 mmol/L; Q2, 1.1–1.6 mmol/L; Q3, 1.6–2.3 mmol/L; and Q4, 2.3–4.3 mmol/L; ^*∗∗∗*^*P* < 0.001, and ^*∗∗*^*P* < 0.01, ^*∗*^*P* < 0.05; Model 1 was not adjusted for variables. Model 2 was adjusted for age, sex, ethnicity. Model 3 was adjusted for age, ethnicity, body mass index, weight, systolic blood pressure, the history of atrial fibrillation, liver cirrhosis, acute myocardial infarction, chronic kidney disease, respiratory failure, chronic obstructive pulmonary disease, ventricular fibrillation, diabetes mellitus, septicaemia, anion gap, bicarbonate, blood urea nitrogen, calcium, serum creatinine, glucose, hematocrit, hemoglobin, platelet, red blood cells, C reactive protein, length of stay, albumin, white blood cells, N terminal pro B type natriuretic peptide, cardiotonic drugs, nitro-glycerine drugs, furosemide drugs, infection-fighting drugs, Simplified Acute Physiology Score II, and sequential organ failure assessment.

**Table 3:** Subgroup analysis for the levels of lactic acid predicting in-hospital mortality.

Variables	Q1	Q2	Q3	Q4	*P* for interaction
COPD					0.201
No	Ref.	1.59 (1.20–2.11) ^*∗∗*^	1.74 (1.31–2.30) ^*∗∗∗*^	2.93 (2.23–3.84) ^*∗∗∗*^	
Yes	Ref.	1.74 (0.89–3.39)	1.93 (0.98–3.80)	1.76 (0.86–3.61)	
DM					0.856
No	Ref.	1.54 (1.16–2.05) ^*∗∗∗*^	1.63 (1.23–2.17) ^*∗∗*^	2.60 (1.97–3.43) ^*∗∗∗*^	
Yes	Ref.	2.02 (1.05–3.86) ^*∗*^	2.44 (1.27–4.69) ^*∗∗*^	3.55 (1.85–6.78) ^*∗∗∗*^	
Hypertension					0.777
No	Ref.	1.64 (1.26–2.15) ^*∗∗∗*^	1.85 (1.42–2.41) ^*∗∗∗*^	2.76 (2.13–3.58) ^*∗∗∗*^	
Yes	Ref.	1.39 (0.48–4.03)	0.51 (0.13–1.96)	2.59 (0.91–7.39)	
RF					0.030
No	Ref.	1.14 (0.73–1.82)	0.97 (0.61–1.57)	2.17 (1.43–3.38) ^*∗∗∗*^	
Yes	Ref.	1.99 (1.44–2.78) ^*∗∗∗*^	2.72 (1.99–3.82) ^*∗∗∗*^	4.66 (3.35–6.57) ^*∗∗∗*^	
Septicaemia					0.133
No	Ref.	1.66 (1.26–2.21) ^*∗∗∗*^	1.73 (1.31–2.30) ^*∗∗∗*^	2.57 (1.96–3.41) ^*∗∗∗*^	
Yes	Ref.	1.47 (0.74–3.04)	1.98 (1.03–4.02) ^*∗*^	3.56 (1.93–7.03) ^*∗∗∗*^	
VF					0.437
No	Ref.	1.79 (1.37–2.37) ^*∗∗∗*^	2.00 (1.53–2.64) ^*∗∗∗*^	2.94 (2.26–3.87) ^*∗∗∗*^	
Yes	Ref.	1.47 (0.74–3.04)	1.98 (1.03–4.02) ^*∗*^	3.56 (1.93–7.03) ^*∗∗∗*^	
AF					0.129
No	Ref.	2.01 (1.35–3.05) ^*∗∗∗*^	1.83 (1.21–2.82) ^*∗∗*^	3.30 (2.24–4.98) ^*∗∗∗*^	
Yes	Ref.	1.25 (0.89–1.76)	1.47 (1.06–2.07) ^*∗*^	2.16 (1.56–3.04) ^*∗∗∗*^	
AMI					0.321
No	Ref.	1.64 (1.24–2.17) ^*∗∗∗*^	1.67 (1.26–2.22) ^*∗∗∗*^	2.54 (1.94–3.36) ^*∗∗∗*^	
Yes	Ref.	1.28 (0.64–2.76)	2.03 (1.02–4.34) ^*∗*^	3.29 (1.69–6.93) ^*∗∗∗*^	
CKD					0.660
No	Ref.	1.59 (1.12–2.31) ^*∗*^	1.67 (1.17–2.42) ^*∗∗∗*^	2.70 (1.91–3.89) ^*∗∗*^	
Yes	Ref.	1.69 (1.17–2.47) ^*∗∗*^	1.93 (1.34–2.83) ^*∗∗∗*^	2.83 (1.99–4.11) ^*∗∗∗*^	

Abbreviation: Q1, 0.3–1.1 mmol/L; Q2, 1.1–1.6 mmol/L; Q3, 1.6–2.3 mmol/L; and Q4, 2.3–4.3 mmol/L; ^*∗∗∗*^*P* < 0.001, ^*∗∗*^*P* < 0.01, and ^*∗*^*P* < 0.05; Model 1 was not adjusted for variables. Model 2 was adjusted for age, sex, ethnicity. Model 3 was adjusted for age, ethnicity, body mass index, weight, systolic blood pressure, the history of atrial fibrillation, liver cirrhosis, acute myocardial infarction, chronic kidney disease, respiratory failure, chronic obstructive pulmonary disease, ventricular fibrillation, diabetes mellitus, septicaemia, anion gap, bicarbonate, blood urea nitrogen, calcium, serum creatinine, glucose, hematocrit, hemoglobin, platelet, red blood cells, C reactive protein, length of stay, albumin, white blood cells, N terminal pro B type natriuretic peptide, cardiotonic drugs, nitro-glycerine drugs, furosemide drugs, infection-fighting drugs, Simplified Acute Physiology Score II, and sequential organ failure assessment.

**Table 4:** Association between NT-proBNP and the risk of in-hospital deaths.

NT-proBNP	Model 1	Model 2	Model 3
Q1, pg/ml	Ref.	Ref.	Ref.
Q2, pg/ml	0.66 (0.44–1.01)	0.62 (0.41–0.95) ^*∗*^	0.40 (0.25–0.64) ^*∗∗∗*^
Q3, pg/ml	1.09 (0.75–1.58)	0.98 (0.67–1.42)	0.71 (0.47–1.07)
Q4, pg/ml	16.01 (12.05–21.27) ^*∗∗∗*^	14.59 (10.96–19.42) ^*∗∗∗*^	13.39 (9.52–18.83) ^*∗∗∗*^
*P* For trend	<0.001	<0.001	<0.001

Abbreviation: NT-proBNP, N terminal pro B type natriuretic peptide; Q1, 122–3589 pg/ml; Q2, 3590–8367 pg/ml; Q3, 8368–11382 pg/ml; Q4, 11383–52201 pg/ml; ^*∗∗∗*^*P* < 0.001, ^*∗*^*P* < 0.05; Model 1 was not adjusted for variables. Model 2 was adjusted for age, sex, ethnicity. Model 3 was adjusted for age, ethnicity, body mass index, weight, systolic blood pressure, the history of atrial fibrillation, liver cirrhosis, acute myocardial infarction, chronic kidney disease, respiratory failure, chronic obstructive pulmonary disease, ventricular fibrillation, diabetes mellitus, and septicaemia, anion gap, bicarbonate, blood urea nitrogen, serum calcium, serum creatinine, glucose, hematocrit, hemoglobin, platelet, red blood cells, C reactive protein, length of stay, albumin, white blood cells, lactic acid, cardiotonic drugs, nitro-glycerine drugs, furosemide drugs, infection-fighting drugs, simplified Acute Physiology Score II, and sequential organ failure assessment.

## Data Availability

The MIMIC-IV database is available in the PhysioNet (https://physionet.org/content/mimiciv/0.4/), and the data can also be obtained from the corresponding authors upon reasonable request.

## Supplementary Materials

Supplementary Materials Supplementary Figure 1: restricted cubic spline plots of associations between lactic acid levels and in-hospital all-cause mortality. Supplementary Table 1: baseline characteristics of the AHF patients on admission. Supplementary Table 2: association between lactic acid levels and the risk of in-hospital mortality.
