# Supplementary material for: Predictive Value of Arterial Blood Lactic Acid Concentration on the Risk of in-Hospital All-Cause Death in Patients with Acute Heart Failure
Source: Int J Clin Pract. 2022 Nov 16;2022:7644535. doi: 10.1155/2022/7644535 (PMC9683964; doi:10.1155/2022/7644535)
Supplement: Supplementary Materials — Supplementary Figure 1: restricted cubic spline plots of associations between lactic acid levels and in-hospital all-cause mortality. Supplementary Table 1: baseline characteristics of the AHF patients on admission. Supplementary Table 2: association between lactic acid levels and the risk of in-hospital mortality. [file 7644535.f1.zip › Supplementary Table 1.docx]

| **Supplementary Table 1**. Baseline Characteristics of the AHF patients on admission. | | | | |
| --- | --- | --- | --- | --- |
| Variables | Overall  (n=322) | Survival group  (n=283) | Death group  (n=39) | P-value |
| Age, years | 69.02 ± 13.39 | 68.13 ± 13.37 | 75.51 ± 11.78 | 0.001 |
| Female, (%) | 143 (44.4%) | 126 (44.5%) | 17(43.6%) | 0.912 |
| BMI, kg/m^2^ | 113.76 ± 15.32 | 114.53 ± 14.30 | 108.21 ± 20.67 | 0.015 |
| Height, cm | 58.32 ± 10.25 | 58.45 ± 9.94 | 57.39 ± 12.40 | 0.546 |
| Weight, kg | 29.63 ± 7.38 | 29.87 ± 7.41 | 27.89 ± 7.00 | 0.117 |
| SBP, mmHg | 168.51 ± 10.62 | 168.47 ± 10.69 | 168.78 ± 10.16 | 0.864 |
| DBP, mmHg | 84.36 ± 22.97 | 84.97 ± 22.95 | 79.93 ± 22.90 | 0.199 |
| AF, (%) | 149 (46.3%) | 128 (45.2%) | 21 (53.8%) | 0.401 |
| Liver cirrhosis, (%) | 6 (1.9%) | 6 (2.1%) | 0 (0.0%) | 0.775 |
| AMI, (%) | 34 (10.6%) | 29 (10.2%) | 5 (12.8%) | 0.832 |
| CKD, (%) | 120 (37.3%) | 101 (35.7%) | 19 (48.7%) | 0.161 |
| Malignancy, (%) | 2 (0.6%) | 1 (0.4%) | 1 (2.6%) | 0.575 |
| RF, (%) | 99 (30.7%) | 74 (26.1%) | 25 (64.1%) | <0.001 |
| COPD, (%) | 39 (12.1%) | 34 (12.0%) | 5 (12.8%) | 0.885 |
| VF, (%) | 4 (1.2%) | 1 (0.4%) | 3 (7.7%) | 0.002 |
| Hypertension, (%) | 20 (6.2%) | 18 (6.4%) | 2 (5.1%) | 0.765 |
| DM, (%) | 62 (19.3%) | 53 (18.7%) | 9 (23.1%) | 0.668 |
| Septicemia, (%) | 32 (9.9%) | 29 (10.2%) | 3 (7.7%) | 0.830 |
| SAP, mmol/L | 14.80 ± 3.64 | 14.53 ± 3.45 | 16.80 ± 4.30 | <0.001 |
| Bicarbonate, mmol/L | 26.41 ± 5.15 | 26.61 ± 5.02 | 24.97 ± 5.84 | 0.062 |
| BUN, mg/dl | 28.50 (19.00, 46.53) | 27.00 (19.00, 44.00) | 36.00 (28.00, 58.50) | 0.003 |
| Calcium, mg/dl | 8.74 ± 0.69 | 8.78 ± 0.66 | 8.51 ± 0.79 | 0.023 |
| Chloride, mmol/L | 100.07 ± 6.09 | 100.07 ± 6.10 | 100.10 ± 6.08 | 0.974 |
| Scr, mg/dL | 1.20 (0.90, 1.80) | 1.20 (0.90, 1.80) | 1.50 (1.00, 2.25) | 0.112 |
| Glucose, mg/dL | 120.00 (100.00, 156.75) | 119.00 (100.00, 155.05) | 122.00 (106.20, 157.50) | 0.203 |
| Sodium, mmol/L | 138.41 ± 4.78 | 138.36 ± 4.82 | 138.74 ± 4.55 | 0.638 |
| Potassium, mmol/L | 4.22 ± 0.56 | 4.20 ± 0.55 | 4.37 ± 0.64 | 0.071 |
| Hematocrit, % | 32.09 ± 6.56 | 32.35 ± 6.68 | 30.20 ± 5.33 | 0.055 |
| Hb, g/dl | 10.32 ± 2.20 | 10.42 ± 2.25 | 9.63 ± 1.67 | 0.036 |
| Plt, ×10^9^/L | 212.00 (148.00, 269.50) | 214.00 (151.00, 271.00) | 181.00 (135.00, 258.00) | 0.155 |
| RBC, ×10^9^/L | 3.55 ± 0.76 | 3.58 ± 0.78 | 3.39 ± 0.62 | 0.157 |
| CRP, mg/L | 48.49 (20.20, 75.62) | 46.04 (18.75, 73.31) | 67.37 (37.13, 97.87) | 0.008 |
| LOS, days | 3.60 (1.99, 6.75) | 3.53 (1.98, 6.59) | 3.85 (2.01, 9.54) | 0.564 |
| Albumin, g/dL | 3.49 ± 0.62 | 3.52 ± 0.61 | 3.22 ± 0.64 | 0.004 |
| Lactic acid, mmol/L | 1.85 ± 0.89 | 1.83 ± 0.88 | 2.63 ± 1.18 | <0.001 |
| WBC, ×10^9^/L | 8.70 (6.90, 11.87) | 8.70 (6.90, 11.80) | 8.80 (8.00, 14.80) | 0.097 |
| NT-proBNP, pg/ml | 8597 (4068, 11762) | 8106 (3753, 10721) | 13833 (11601, 16122) | <0.001 |
| Cardiotonic, (%) | 86 (26.7) | 71 (25.1) | 15 (38.5) | 0.115 |
| Nitroglycerin, (%) | 157 (48.8) | 145 (51.2) | 12 (30.8) | 0.026 |
| Furosemide, (%) | 312 (96.9) | 275 (97.2) | 37 (94.9) | 0.776 |
| Hypoglycemic, (%) | 62 (19.3) | 54 (19.1) | 8 (20.5) | 0.488 |
| Infection-fighting, (%) | 39 (12.1) | 26 (9.2) | 13 (33.3) | <0.001 |
| SAPS II score | 40 (33, 50) | 40 (33, 48) | 49 (38, 58) | 0.002 |
| SOFA score | 7 (5, 12) | 7 (5, 11) | 11 (7, 14) | <0.001 |

Abbreviations: AHF, Acute Heart failure; BMI, body mass index; SBP, systolic blood pressure; DBP, diastolic blood pressure; AF, atrial fibrillation; AMI, acute myocardial infarction; CKD, chronic kidney disease; RF, respiratory failure; VF, ventricular fibrillation; SAP, Serum anion gap; BUN, blood urea nitrogen; SCr, serum creatinine; Hb, Hemoglobin; Plt, Platelet; RBC, red blood cell; CRP, C-reactive protein; SOFA, sequential organ failure assessment. LOS, length of stay; WBC, white blood cell; NT-proBNP, N terminal pro B type natriuretic peptide; SAPS II score, Simplified Acute Physiology Score II.
