# Supplementary material for: Predictive Value of Arterial Blood Lactic Acid Concentration on the Risk of in-Hospital All-Cause Death in Patients with Acute Heart Failure
Source: Int J Clin Pract. 2022 Nov 16;2022:7644535. doi: 10.1155/2022/7644535 (PMC9683964; doi:10.1155/2022/7644535)
Supplement: Supplementary Materials — Supplementary Figure 1: restricted cubic spline plots of associations between lactic acid levels and in-hospital all-cause mortality. Supplementary Table 1: baseline characteristics of the AHF patients on admission. Supplementary Table 2: association between lactic acid levels and the risk of in-hospital mortality. [file 7644535.f1.zip › Supplementary Table 2.docx]

| **Supplementary Table 2.** Association between lactic acid levels and the risk of in-hospital deaths. | | | |
| --- | --- | --- | --- |
| Lactic acid | Model 1 | Model 2 | Model 3 |
| Q1, mmol/L | Ref. | Ref. | Ref. |
| Q2, mmol/L | 1.23 (0.63, 2.41) | 1.22 (0.62, 2.41) | 1.23 (0.57, 2.64) |
| Q3, mmol/L | 1.64 (0.87, 3.11) | 1.58 (0.83, 3.01) | 1.82 (0.89, 3.72) |
| Q4, mmol/L | 2.54 (1.36, 4.76) ** | 2.59 (1.37, 4.89) ** | 2.68 (1.27, 5.63) ** |
| *P* for trend | < 0.001 | < 0.001 | < 0.001 |

Abbreviation: Q1, 0.5-1.2 mmol/L; Q2, 1.3-1.6 mmol/L; Q3, 1.7-2.4 mmol/L; Q4, 2.5-4.7 mmol/L; ***P* < 0.01; Model 1 was not adjusted for variables. Model 2 was adjusted for age, sex. Model 3 was adjusted for age, sex, body mass index, the history of respiratory failure, and ventricular fibrillation, serum anion gap, blood [urea nitrogen](javascript:;), serum calcium, hemoglobin, C reactive protein, albumin, N terminal pro B type natriuretic peptide, nitroglycerin drugs, infection-fighting drugs, simplified Acute Physiology Score II, and sequential organ failure assessment.
