# Supplementary figures and images for: Predictive Value of Arterial Blood Lactic Acid Concentration on the Risk of in-Hospital All-Cause Death in Patients with Acute Heart Failure
Source: Int J Clin Pract. 2022 Nov 16;2022:7644535. doi: 10.1155/2022/7644535 (PMC9683964; doi:10.1155/2022/7644535)

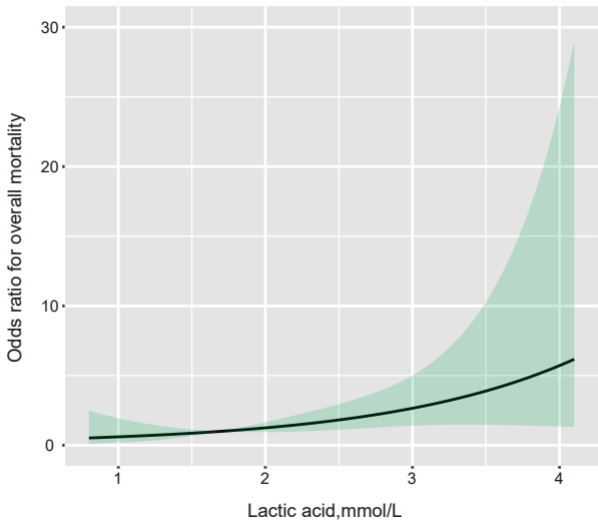

Supplement: Supplementary Materials — Supplementary Figure 1: restricted cubic spline plots of associations between lactic acid levels and in-hospital all-cause mortality. Supplementary Table 1: baseline characteristics of the AHF patients on admission. Supplementary Table 2: association between lactic acid levels and the risk of in-hospital mortality. [file 7644535.f1.zip › Supplementary Figure 1.pdf]
